# Characteristics of a Nationwide Voluntary Antibiotic Resistance Awareness Campaign in India; Future Paths and Pointers for Resource Limited Settings/Low and Middle Income Countries

**DOI:** 10.3390/ijerph16245141

**Published:** 2019-12-16

**Authors:** Ashok J. Tamhankar, Ramesh Nachimuthu, Ravikant Singh, Jyoti Harindran, Gautam Kumar Meghwanshi, Rajesh Kannan, Nachimuthu Senthil Kumar, Vikrant Negi, Lijy Jacob, Sayan Bhattacharyya, Krushna Chandra Sahoo, Vijay Kumar Mahadik, Vishal Diwan, Megha Sharma, Ashish Pathak, Smita U. Khedkar, Dnyaneshwar Avhad, Sonal Saxena, Sandeep Nerkar, Vaishali Venu, Sandeep Kumar, G. Shandeepan, Khundrakpam Ranjit Singh, Ridiamma Gashnga, Arvind Kumar

**Affiliations:** 1 Indian Initiative for Management of Antibiotic Resistance, 302, Aryans, Deonar, Mumbai 400088, India; 2 Department of Public Health Sciences, Karolinska Institutet, 171 77 Stockholm, Sweden; vishal.diwan@ki.se (V.D.); meghasharma27@rediffmail.com (M.S.); drashishp@rediffmail.com (A.P.); 3 Indian Initiative for Management of Antibiotic Resistance, Antibiotic Resistance and Phage Laboratory, Vellore Institute of Technology, Vellore 632014, India; drpnramesh@gmail.com; 4 Chief Functionary’s Office, Doctors For You, Lallubhai Compound, Mankhurd, Mumbai 400 043, India; ravikant.singh@doctorsforyou.org; 5 Departmentof Pharmaceutical Sciences, Centre for Professional and Advanced Studies, Cheruvandoor Campus, Ettumanoor, Kottayam, Kerala 686631, India; jharindran@yahoo.com; 6 Department of Microbiology, Maharaja Ganga Singh University, NH-15, Jaisalmer Road, Bikaner, Rajasthan 334 001, India; drgkm_biotech@yahoo.com; 7 Department of Microbiology, Bharathidhasan University, Thiruchirapalli, Tamilnadu 620024, India; uvrajesh@gmail.com; 8 Department of Biotechnology, Mizoram University, Aizwal, Mizoram 796004, India; nskmzu@gmail.com; 9 Department of Microbiology, Dr. S.N. Medical College, Jodhpur, Rajasthan 342 001, India; negi.vikrant@gmail.com; 10 Department of Biotechnology, St. Berchmans College, Changanassery, Kerala 686101, India; lijyjinu@gmail.com; 11 Department of Microbiology, All India Institute of Medical Sciences, Patna, Bihar 801507, India; sayantheboss@yahoo.co.in; 12 Department of Health Research, ICMR-Regional Medical Research Centre, Bhubaneswar, Odisha 751023, India; sahookrushna@yahoo.com; 13 Department of Public Health and Environment, R.D Gardi Medical College, Ujjain, Madhya Preadesh 456006, India; uctharc@sancharnet.in; 14 Medical Director’s office, Department of Public Health and Environment, R.D Gardi Medical College, Ujjain, Madhya Preadesh 456006, India; 15 Department of Pharmacology, R.D Gardi Medical College, Ujjain, Madhya Preadesh 456006, India; 16 Department of Paediatrics, R.D Gardi Medical College, Ujjain, Madhya Preadesh 456006, India; 17 Bactest Laboratory and Dental College, Nashik, Maharashtra 422 005, India; udayk_nsk@sancharnet.in; 18 School of Health Systems Studies, Tata Institute of Social Sciences, Mumbai 400088, India; drdna1990@gmail.com; 19 Department of Microbiology, Lady Hardinge Medical College, Delhi 110 001, India; sonalsaxena3@gmail.com; 20 Chetana Laboratories, Nashik, Maharashtra 422009, India; san_ner@rediffmail.com; 21 Director-Health services’ offce, Doctors For You, Lallubhai Compound, Mankhurd, Mumbai, Maharashtra 400043, India; vaishali.dfy@gmail.com; 22 Doctors For You, Patna, Bihar 803 201, India; sandeep.dfy@gmail.com; 23 Doctors For You, Bandipore, Jammu and Kashmir 193502, India; shandeepang@doctorsforyou.org; 24 Doctors For You, Ningthoukhong Ward No.3, Bishnupur District, Manipur 795126, India; ranjit.dfy@gmail.com; 25 Doctors For You, Laitumkhrah Nongrim Road, Shillong, Meghalaya 793003, India; ridiamagashnga19917@gmail.com; 26 Doctors For You, A-58, Plot no. 7, Block A extension, Budh Vihar, Delhi, Budh Vihar, Delhi 110086, India; dr.arvind.dfy@gmail.com

**Keywords:** antibiotic resistance, electro–physical awareness campaign, pedagogical and participatory techniques, India, community radio stations, campaign characteristics

## Abstract

Antibiotic resistance has reached alarming proportions globally, prompting the World Health Organization to advise nations to take up antibiotic awareness campaigns. Several campaigns have been taken up worldwide, mostly by governments. The government of India asked manufacturers to append a ‘redline’ to packages of antibiotics as identification marks and conducted a campaign to inform the general public about it and appropriate antibiotic use. We investigated whether an antibiotic resistance awareness campaign could be organized voluntarily in India and determined the characteristics of the voluntarily organized campaign by administering a questionnaire to the coordinators, who participated in organizing the voluntary campaign India. The campaign characteristics were: multiple electro–physical pedagogical and participatory techniques were used, 49 physical events were organized in various parts of India that included lectures, posters, booklet/pamphlet distribution, audio and video messages, competitions, and mass contact rallies along with broadcast of messages in 11 local languages using community radio stations (CRS) spread all over India. The median values for campaign events were: expenditure—3000 Indian Rupees/day (US$~47), time for planning—1 day, program spread—4 days, program time—4 h, direct and indirect reach of the message—respectively 250 and 500 persons/event. A 2 min play entitled ‘Take antibiotics as prescribed by the doctor’ was broadcast 10 times/day for 5 days on CRS with listener reach of ~5 million persons. More than 85%ofcoordinators thought that the campaign created adequate awareness about appropriate antibiotic use and antibiotic resistance. The voluntary campaign has implications for resource limited settings/low and middle income countries.

## 1. Introduction

Antimicrobial resistance (AMR) is the ability of a microorganism (like bacteria, viruses, and some parasites) to stop an antimicrobial (such as antibiotics, antivirals, and antimalarials) from working against it and as a result, standard treatments become ineffective, infections persist, and may spread to others [1]. Resistance development is an evolutionary phenomenon, although mutations can also contribute to it. Some known resistance mechanisms are: decreased cell permeability, active efflux pump, enzymatic inactivation, modification of drug receptor site, and synthesis of resistant metabolic pathways by microbes. Human actions such as inappropriate use of antibiotics, poor infection prevention and control etc., magnify resistance. In general, AMR, particularly, antibiotic resistance, is on the rise worldwide.

India has a three-tier healthcare system (primary, secondary, tertiary), which has both public and private healthcare players, and is manned by allopathic as well as AYUSH (Ayurvedic, Unani, Siddha, Homeopathic—the non-allopathic systems of medicine practiced in India) practitioners, with all of them potentially prescribing antibiotics. In addition, informal healthcare providers (healthcare providers who have not received a formal degree in medicine from any institution and are not registered as healthcare practitioners with any governing body) and pharmacists may also prescribe/dispense antibiotics, with antibiotics likely to be also available ‘over the counter’ without prescription on demand by lay persons [2,3,4]. The government has created laws and policies regarding antibiotic use, but their enforcement is still weak. Thus, there is a large scope for conducting antibiotic/antibiotic resistance awareness programs/campaigns in India to educate various stakeholders, particularly students—the future stakeholders—about appropriate use of antibiotics and infection prevention and control.

The World Health Organization (WHO) published a global report on antimicrobial resistance surveillance in 2014 emphasizing that “resistance in common bacteria has reached alarming levels in many parts of the world and that in some settings, few treatment options remain effective for common infections” [5]. It was estimated in another report in 2014 that if no action is undertaken to curb antimicrobial resistance, nearly 10 million deaths may occur by 2050 due to causes related to antimicrobial resistance [6]. Considering the alarming situation, the WHO came up with a global action plan on antimicrobial resistance in 2015 to be modified and implemented by all the member nations [7]. India came up with its National Action Plan (NAP) in April 2017 [8]. In August 2017, the Indian government organized a meeting to operationalize the Indian NAP, where the Indian Initiative for Management of Antibiotic Resistance (IIMAR) was invited to participate through IIMAR’s national coordinator [9].Further, in 2017, the WHO advised its member states to undertake antibiotic awareness campaigns during 13–19 November 2017 and also suggested certain materials to be used as campaign components [10].A recent systematic review (2019) informs that public awareness campaigns and antimicrobial guidelines are the most commonly used policy strategies for potentially effecting appropriate antimicrobial use and reducing antimicrobial resistance [11]. The review found 17 communication interventions i.e., campaigns made through print, electronic, telephonic, or broadcast media globally and informs that these were government initiatives implemented by governments, government aided bodies with government aid/funding. In India, the government appended a ‘redline’ to the packages of antibiotics as identification marks and conducted a nationwide campaign to inform the general public about it and appropriate antibiotic use. In general, information is lacking on whether large scale (dimensions similar to India), nationwide antibiotic/antibiotic resistance awareness campaigns can be conducted successfully on a ‘voluntary’ basis without government aid/funding, where ‘voluntary’ means people volunteer their time and money to the cause without government support and outside of their typical jobs. In view of this, IIMAR, a zero funded voluntary organization, which had earlier conducted small size programs voluntarily to raise awareness about antibiotics/antibiotic resistance related issues, decided to investigate whether a nationwide campaign could be conducted in India on raising awareness about prudent antibiotic use, escalating antibiotic resistance, and maintenance of hand hygiene on a voluntary participation basis by IIMAR-associated individuals. It was also decided to investigate the characteristics of such a campaign.

This paper reports the results and characteristics of a campaign conducted in India on a voluntary initiative, entitled ‘Antibiotic Resistance Awareness Campaign (AMRAC-17)’ during 13–19 November 2017, and discusses future paths and pointers for resource-limited settings/low and middle income countries. In the article, the terms antimicrobial and antibiotic appear synonymously.

## 2. Materials and Methods

### 2.1. Setting

India is a country of sub-continental dimensions (Area—3287,590 km^2^, population nearing 1.37 billion) [12], divided into six zones—north, north east, east, south, west, and central zone—that include 28 states and 9 union territories. It was proposed to conduct AMRAC-17in as many places as possible, in all the six zones of India. 

### 2.2. Campaign Participation

A national level committee of Ashok J. Tamhankar (A.J.T.), National Coordinator of IIMAR, Ramesh Nachimuthu (R.N.), Joint Coordinator of IIMAR, and Ravikant Singh (R.S.), Chief Functionary of Doctors For You (DFY) coordinated the campaign. IIMAR and DFY are both non-governmental organizations (NGO) working in the field of public health. While IIMAR devotes itself mainly to management of antibiotic resistance, DFY works in the entire public health field, including disaster relief. Both have a wide network of non-healthcare and healthcare-related individuals interested in the objectives of these organizations throughout India. The potential campaigners, who were in these organization’s connectivity range–network and also some who became interested by reading the IIMAR blog and Facebook page, were invited to join the campaign through phone calls, emails, webpages, Facebook page, and ‘WhatsApp’ group (Antibiotic Resistance Ind) of IIMAR. Those who accepted the invite were sent a form to fill in with their details [13]. Once recruited, a ‘WhatsApp’ group tool “AMR week 2017 campaign” was also employed for further coordination. The campaigners were sent general instructions regarding how to conduct the program (Supplementary DocumentS1) and the campaign materials electronically.

### 2.3. Campaign Characteristics

We planned to use multiple pedagogical and participatory physical and electronic techniques such as display of posters, distribution of pamphlets/booklets, audio and video messages, radio broadcast, lectures, slogan competition, rallies, etc., for the dissemination of the campaign message.

The posters in English made available to coordinators were the ones advised by WHO [10]. The posters made available in Hindi, the national language of India, and local languages were based on the WHO material. Pamphlets and booklets were created indigenously at both national and local levels. An audio message—a two minute duration play—was made available in 11 languages (Bengali, Dogri, English, Gujarati, Hindi, Kannada, Marathi, Malayalam, Odiya, Tamil, and Telugu) for broadcast during AMRAC-17 from community radio stations (CRS) spread all over India. Video messages showing how to maintain hand hygiene (HH) with soap and water and with an alcohol-based hand rub (ABHR) were made in English and Hindi. The pre-testing/validating of the non-WHO campaign material was done within the coordinating group.

To understand the characteristics of the campaign, after the campaign was over, the coordinators were asked to respond to a questionnaire (Supplementary DocumentS2) that elicited information on the coordinator’s demographic information and monetary, personnel, duration, experience, success, utility, outreach, behavior change aspects of the campaign. The data was entered in Excel and descriptive statistics were used for presentation of results.

IIMAR did not provide any funding or incentive to the coordinators and the volunteers. They were to fund their activity on their own.

## 3. Results

### 3.1. Campaign Participation and Spread

As a response to our call, 69 potential collaborators showed interest and were sent the form to register [13]. To this, 42 responded and were sent the campaign material and instructions about how to conduct the program (Supplementary Document S1). The post-campaign questionnaire (Supplementary DocumentS2) was sent to all these. In spite of repeated reminders, only 19 responded and they/their team and the campaign coordinating team have been included as authors. The paper reports only the contributions of these.

The campaign message encompassed in a 2 min play entitled ‘Take antibiotics as prescribed by doctors’ (Supplementary audio S1) was broadcast over CRS spread all over India 10 times a day for 5 days during the campaign.

**Figure 1:**
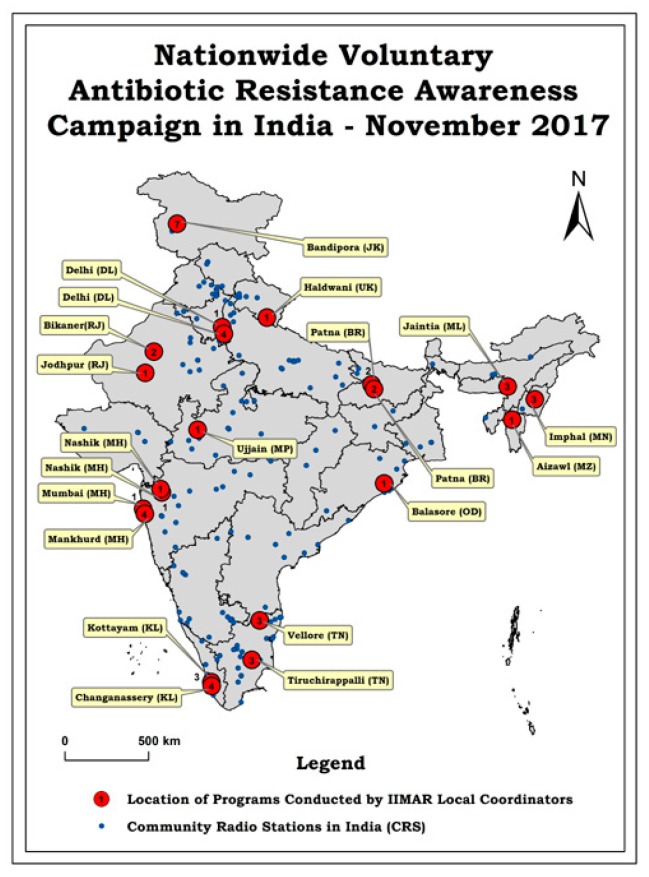
Map of India showing locations where the Antibiotic Resistance Awareness Campaign conducted during 13–19 November 2017 (AMRAC-17) by the Indian Initiative for Management of Antibiotic Resistance (IIMAR) coordinators and the community radio stations occurred. Numbers along with large circles indicate the number of events/programs the coordinators organized at the mentioned and nearby locations.

The extent of the campaign’s geographical spread, inclusive of physical campaign places and CRS, can be visualized in Figure 1.

### 3.2. Campaign Components

**Figure 2:**
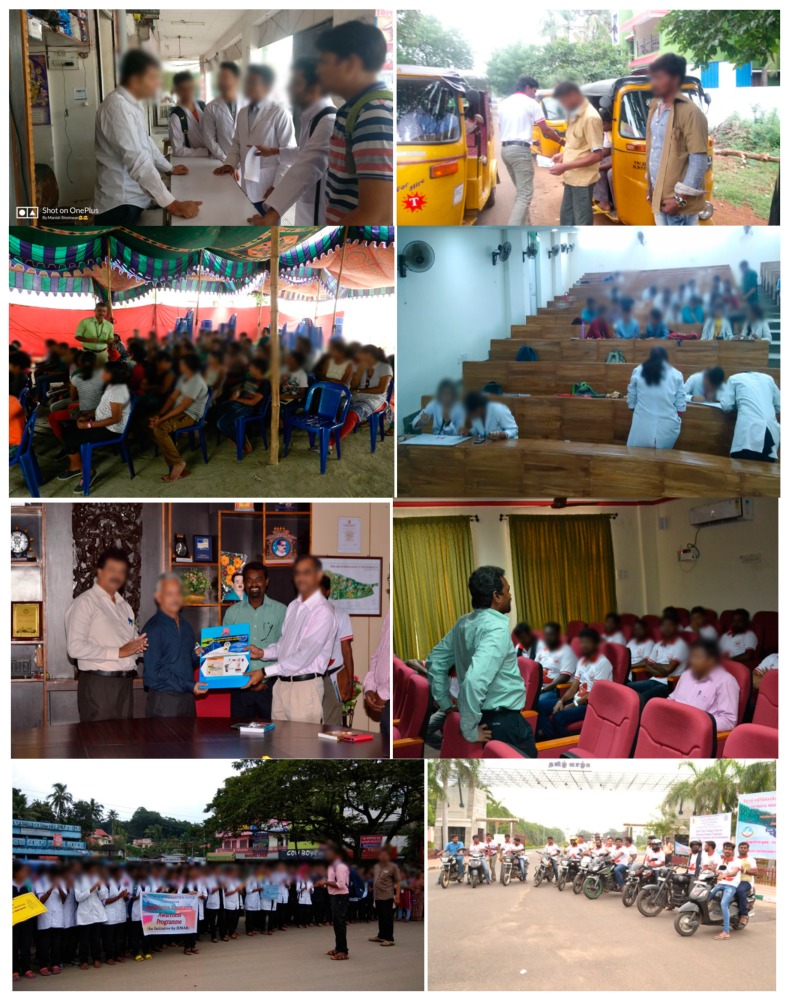
Examples of mass public contact during AMRAC-17 in India (clockwise, starting from upper left)—campaigners with pharmacist, auto-riksha drivers, medical college students (poster/slogan competition), science college students, two wheeler rally, pharmacy students rally, offices, school students.

**Figure 3:**
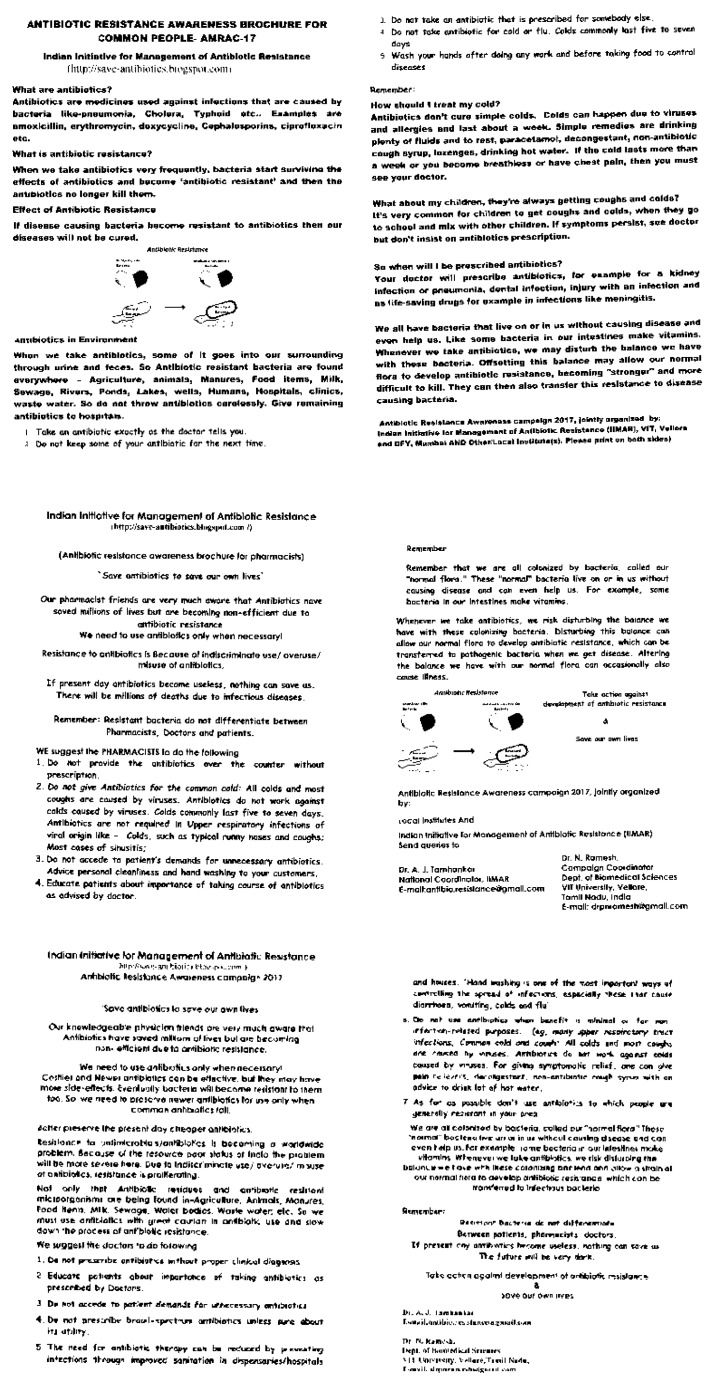
Single page pamphlets distributed during AMRAC-17 in India (front and back side) among general public (first row), pharmacists (second row), physicians (third row).

The text of the invitation to potential collaborators for the nationwide campaign is available at the IIMAR webpage [14]. Mass contact by poster display, pamphlet distribution, office visits, school/college lectures, poster making/slogan writing competitions, and rallies were organized locally to create awareness about prudent use of antibiotics and antibiotic resistance (Figure 2). Some examples of various posters used and examples from poster making and slogan writing competitions are presented in Supplementary Figure S1. Single page pamphlets that were distributed to the general public, pharmacists, and physicians/healthcare workers are presented in Figure 3. A sample of the Tamil language version of these and other examples of English and local language foldable pamphlets are available in Supplementary Figure S2.

An example of an audio message in English entitled ‘Take antibiotics as prescribed by the doctor’ broadcasted on CRS and circulated among the public is presented in Supplementary audios S1–S5. The video messages in English and Hindi entitled ‘Prevent the development of antibiotic resistance and spread of infections by hand hygiene’ described what are antibiotics, what is antibiotic resistance, what are its implications, how to prevent antibiotic resistance, what is prudent antibiotic use, and how to prevent infections by following hand hygiene and the method of hand hygiene. The English version is available in Supplementary video S1.

### 3.3. Campaign Characteristics

Table 1 indicates campaign characteristics that emerged from the responses of the coordinators to the post-campaign questionnaire.

Among the coordinators, 48% were PhDs, 12% were doctors of medicine, 32% had Master’s degrees, 8% had Bachelor’s degrees. The male: female ratio among coordinators was 72:28. The coordinator’s specializations included medicine, public health, international/global health, pharmacy, disaster management, microbiology, biochemistry, biotechnology, dentistry, nursing, environmental health, limnology, and social work. The type of institutions/organizations they belonged to included: educational institutes-private—9, government—5, (among these 5 were teaching medical colleges), NGO—2, private pathological laboratories—2. Some coordinators organized more than one distinct campaign event each: three organized 4 events, 5 organized 3 events, 3 organized 2 events, and the rest organized one event each (Figure 1). In Bandipore district in Jammu and Kashmir, from the campaign week onwards, the campaign contents were included in the health awareness lectures for one year. However, it has been considered as 7 events (campaign week). The CRS broadcast of the 2 min play was for 10 times a day for 5 days. In the case of 75% of the coordinators, the funds were offered by their institutions. For others, it was volunteer’s donations at 15% and coordinator’s contribution at 10%.

The distribution of the locations of the campaign was: city 46%, district place 27%, villages 27%. The campaign in Jammu and Kashmir covered several villages in the Bandipore district. CRS in India were distributed among educational institutes (schools, colleges, universities)—57% and NGOs—43%. While the educational institute’s campuses were generally away from cities and were self contained, the NGO’s CRS generally focused on rural and remote areas.

**Figure 4:**
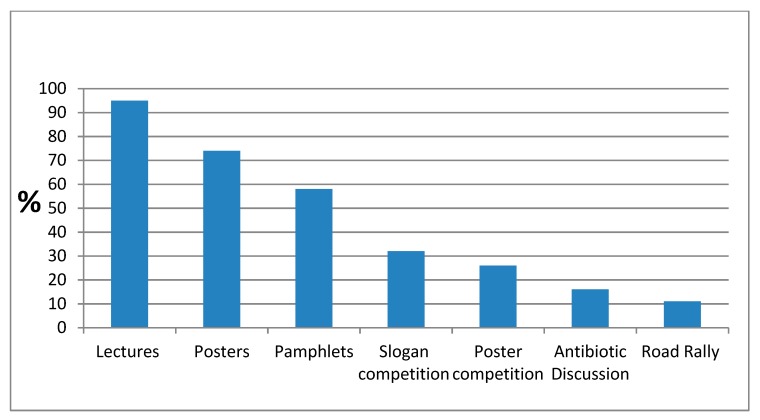
Top preferences for individual campaign components by campaign coordinators during AMRAC-17 in India. Notes: (1) In addition to these components, the other components the coordinators also used were: LED display, amphitheater display, essay competition, social media, souvenir, awards, interactive discussion on antibiotics, role play for medical and nurse students, radio broadcast, display of wrappers of antibiotics. (2) All the earlier mentioned components (Figure 4+ Note (1)) were used by coordinators in various combinations according to their convenience and imagination, with 21% coordinators using only lectures in their campaign. The distribution of the target audience for the campaign events was, lay public 48.4%, healthcare professionals 22.6%, healthcare students 16.2%, non-healthcare students 12.8%. The lectures took place in class rooms/auditoriums of educational institutes, NGO’s class rooms/waiting halls/office, village Panchayat (village governing council) halls/rooms.

The campaign components preferred by the coordinators can be seen in Figure 4. Lectures were the most preferred item (coordinator—68%, invited guest—42%), followed by posters (made locally—50%, supplied by IIMAR—50%), pamphlets (made locally—42%, supplied by IIMAR 48%). The distribution of other components is explained in Figure 4. It was presumed that the audio and video made by IIMAR were utilized by the coordinators in the campaign and also through WhatsApp.

Table 2 indicates some more responses from the coordinators to the post campaign questionnaire. The main difficulty faced by the coordinators was absence of centralized funding and the main comment was, if the government had funded the program, it would have been more extensive and more successful. The other comments with regards to difficulties faced were (in coordinator’s words)—‘To reach to the level of recipient’s understanding and to re-instill new information’, ‘making people aware that missing a dose can lead to resistance’, ‘to educate people about the concept of antibiotic resistance’, ‘creating awareness about prudent use of antibiotics’, ‘to convince ‘quacks’ (In India, in some places totally untrained village doctors also prescribe allopathic medicine.) [2] about appropriate use of antibiotics.’

## 4. Discussion

### 4.1. Campaign Components and Characteristics

The salient feature of our campaign was that the campaigners could choose from a catalogue of electro-physical (electronic and physical) campaign materials, whatever was feasible for them to utilize. Lectures appeared to be the most preferred item. The campaigners and the organizations at which and through which the campaign was conducted, and the recipients of the campaign, included healthcare-related and non-healthcare-related stakeholders, students and non-students. The geographical spread of the campaign was throughout the country. More than 80% of coordinators perceived that the campaign delivered the targeted messages.

A covariate analysis had earlier provided theoretical insight into the kind of groups to be targeted for antimicrobial awareness campaigns, namely, stewards, stockers, and demanders [15]. The analysis indicated that campaigns should encourage stewards-healthcare providers to follow their intentions regarding prescribing/not prescribing antibiotics, encourage stockers to not to stock or share their leftover antibiotics, and convince demanders to accept a healthcare provider’s evidence-based judgment regarding antibiotic prescription. Our campaign material and programs followed this dictum; see particularly, the pamphlets directed towards general public, prescribers, and pharmacists (Figure 3). This approach also helped antimicrobial stewardship, which is defined as “the optimal selection, dosage, and duration of antimicrobial treatment that results in the best clinical outcome for the treatment or prevention of infection, with minimal toxicity to the patient and minimal impact on subsequent resistance” [16].

Another suggested approach for a campaign is to adopt a social marketing approach to achieve sustainable behavior changes. Social marketing is a ‘process based on the application of marketing principles and techniques to create, communicate, and deliver social values designed to influence target audience behavior so that both society and the target audience benefit according to the ideological framework used’ [17]. Since we used WHO-advised posters and the key message advised for 2017, “Take antibiotics only with the advice of doctor”, we believe that WHO must have kept this in view while developing the posters and the key message. In our campaign, we also added importance of ‘hand hygiene’ to it, bringing into focus the ‘risk to life’ factor associated with hand contamination leading to contacting infectious diseases. Further, India is a multilingual country, so we provided a catalogue of campaign materials in several languages, from which the coordinators could choose whatever they found suitable locally. Thus, we tried to give a product mix and provided a flexible promotion strategy aimed at the stakeholders to ‘own’ the campaign message.

The approach is illustrated by taking an example of the audio and video messages that we created. The audio message, which was in 13 languages, (for the English version, refer to Supplementary audio S1) started with misuse of antibiotics in cases of cough and cold and lead the listener to the misuse in cases of loose-motions and viral infections and where antibiotic use is appropriate or inappropriate and that antibiotics should only be taken with the advice of a doctor. The audio had three actors, a parent (male/female), a child (male/female), and the doctor (male/female). By incorporating the three components of the relevant societal set up, we made the appeal contextually universal. We made the child to ask the doctor why and when the doctor gives antibiotic treatment. Thus, we kept the respect of the family head—the parent—and also ensured that the children, the future generations, become interested in the message. The incorporation of the doctor in the audio gave authority to the message and also ownership and advice to prescribing healthcare workers as to when they should prescribe antibiotics. The context to this is that in India, healthcare providers, both trained and untrained in allopathic medicine, are likely to prescribe antibiotics [2,18]. So, such a message gave them appropriate advice without hurting the prescriber’s feelings. For the video message also, we had a similar approach. Four videos were created, two in English and two in Hindi, one each for hand washing with soap and water and one for ABHR. The videos were enacted by a female expert, (refer to Supplementary video S1) who first established her credentials as being from a medical college, described her expertise in the field of antibiotics and hand hygiene, remarked about the importance of hand hygiene in the context of the campaign, and then demonstrated how to maintain hand hygiene with soap and water or ABHR. For hand wash and hand rub, it was important to have the presentation in video format to demonstrate the correct method of maintaining hand hygiene.

We also used ‘community radio stations (CRS)’ to spread the campaign message. Community radio has been defined as “one that is operated in the community, for the community, about the community and by the community-the community can be territorial, or geographical e.g., township, village, district, or island” [19]. CRS are rooted in the local community and focus on local development goals for health, nutrition, education etc. CRS in India broadcasts in local languages and the two official languages of national communication—Hindi and English. CRS have a distinct advantage in a country like India, where in various geographical regions varying languages are spoken and messages delivered in local languages become an ideal instrument of positive change and community empowerment. At the time of the campaign, there were 238 CRS distributed among educational institutes—57% and NGOs—43% [20]. (This number keeps changing, both because of operational issues and issues regarding licenses.) A listenership survey for 200 CRS reported the following key findings [21]: (a) estimated listener households ~4.77 million; (b) 93% households listen to radio 3–4 times a week; (c) 82% listeners perceived that health/hygiene programs were useful; (d) 62% perceived that programs related to disease prevention were useful. (Average of d + e = 72%); (e) 77% hail from rural areas. From this, if we consider that 70% households found our health related message useful, the reach of the message could be estimated to be about 3.33 million households. The average number of persons/household in India is 4.8 [22]. An average of 2 of these members listening to our message takes the listenership for our message potentially to >5 million, out of which >4 million may be in rural areas. Considering this outreach, CRS can be looked upon as a potent communication tool for campaigns related to antibiotic/antibiotic resistance awareness.

A campaign is a planned set of activities to achieve a certain objective [23]. In the current context, they are activities conducted to change human behavior towards antibiotics. Theories of human behavior in the context of antibiotic use, factors influencing behavior towards antibiotics and approaches to change antibiotic related behavior have been discussed earlier [24]. It is stated therein that ‘providing correct knowledge is a pre-requisite for behavior modification in the desired direction; although, we can never change the behavior of any other human, we can facilitate for others to change their own behavior’. We believe that our campaign and its components facilitated this objective, a change in antibiotic related behavior.

Antimicrobial/antimicrobial resistance awareness campaigns have been conducted earlier [25], generally by governments/government aided bodies/international agencies with their funding, e.g., campaigns in France [26], Sweden [27], United Kingdom [28], Greece [29], Belgium [30], Thailand [31], India-Red-Line campaign [32], New Zealand [33], Australia [34], and at a continental level in Europe [35]. Antimicrobial/antimicrobial resistance awareness campaigns by voluntary action and on voluntary funding basis are rare or not very known and ours must be a rare example of such a campaign. Campaigns aimed to promote prudent use of antibiotics and reduce resistance levels have been reported to achieve varying degrees of effectiveness [36,37,38,39,40,41] with some reports indicating success in reduction in antibiotic use [37,40], while others indicating that they may not be always successful [41]. A view exists that there are conceptual, methodological, and empirical weaknesses to the campaign approach [42] However, recommendations to conduct campaigns aimed at changing antibiotic-related behavior and thus control antibiotic resistance continue [10,36,43], indicating a perception that such campaigns have a desired impact on antibiotic-related issues.

Considering the complexity and diversity of India, our campaign setting was somewhat comparable to the campaign setting in the European Union (EU) [35], although ours was a voluntary campaign while the EU campaign was backed by governments. The EU is a union of 28 member states, with an area of 4.476 million km^2^, an estimated population of about 513 million, with several spoken languages and a variety of cultures [35]. Information about India provided earlier would indicate the similarities between the two. One of the differences is that a majority of the countries in EU are high income countries (HIC), while India is a lower middle income country (LMIC). While a scoping report [44] and the Chennai Declarations [45] put forth the antibiotic-related situation in India succinctly, some estimates of antibiotic consumption for India are available, such as 10,608 standard units per 1000 population in 2010 (one standard unit = number of doses sold in the country; a dose being a pill, capsule, or ampoule) [46], 16.0 DDD per 1000 individuals in 2012 (DDD = daily defined dose) [47] and 11,597 standard units per 1000 population in 2014 [48]; however, community level antibiotic consumption rates in India are still low as compared to European Surveillance of Antimicrobial Consumption Network (ESAC-Net) countries (16.0 DDD vs. 21.54 DDD per 1000 individuals, 2012 estimate) [47] and thus the antibiotic consumption in India is likely to increase. Therefore, for India and similar countries, if antibiotic/antibiotic resistance awareness campaigns are taken up right earnestly, there could be an outcome in terms of appropriate use of antibiotics and reduction in resistance. Thus, our campaign and its methodology was a step taken in the right direction.

Since ours was a first attempt for a nationwide voluntary campaign in India, we compare here our campaign characteristics with the first EU campaign in 2008 [35]. An analysis of the EU campaign indicated that the visuals were used in a wide array of materials: posters (63%), information leaflets (47%), web pages (53%), letters (44%), advertisements (28%), and TV spots (16%). This information regarding our campaign is given in Figure 4. We had IIMAR webpages [14] and Facebook pages that are all-time running campaigns and always accessible. Instead of letters, we used ‘WhatApp’ group as well as personal ‘WhatsApp’ accounts for spreading the messages. There were no advertisements and TV spots in our campaign, as we did not have funds for these. However, we used the community radio stations with a very wide outreach to spread the audio message. Lectures were not a part of EU campaign. In almost all of our events, lectures were organized. Lectures leading to personal interaction are also cheaper, as an individual lecturer is a sufficient resource, and if a volunteer, no expense is involved.

Some of the EU responses pointed out that the teams involved in the EU campaign were handling this campaign in addition to their regular work. This was true for us also. The respondents in the EU campaign indicated that their governments supported the campaign politically and financially. We did not have any support from government for our effort. Earlier information on budgets and funding reflected widely varying volume and intensity, from a budget of a few thousand USD, to expensive mass-media campaigns with budgets in the range of millions of USD [22]. Ours was a voluntary campaign and the maximum expenses reported were 775 USD with a median of 47 USD. The median expense programs had the common feature of distribution of pamphlets and display of posters with the addition of lectures or listening to audio and discussion on antibiotics. Further, for LMICs with limited resource settings, the addition of CRS broadcast could be the way for spreading antibiotic/antibiotic resistance awareness and effect resistance reduction.

The UK “Antibiotic Guardian Campaign” is an ‘always on’ campaign for AMR [49] The IIMAR web pages [10] and Facebook pages can be considered as an ‘always on’ campaign. A policy frame analyses of AMR identified that AMR fitted into frames such as healthcare, development, innovation, security, and One Health, finding each frame originating in distinct scientific fields and recommending integration of these frames into an overarching social and ecological framework for policy progress in tackling AMR [50]. Indeed, probably campaigns hereafter should develop an integrated approach, taking into consideration all these frames to have an effective outcome.

This is probably the first and only antibiotic resistance awareness campaign conducted on such a wide scale (geographical and multilingual spread) on a voluntary basis using electronic and physical campaign components in India and also probably, globally. We did not come across any reference in the literature for a comparable voluntary, non-centrally funded, wide scale campaign on antibiotic resistance awareness and so, our campaign becomes a significant achievement.

Organizing public awareness campaigns in a multicultural, multilingual, and vast country like India on a voluntary basis is a challenge, particularly to take along a lot of un-acquainted, faceless, unknown people, bound only by a desire to reduce antibiotic resistance and promote appropriate antibiotic use. Absence of centralized funding was a great impediment. There was a general consensus among coordinators that if government or other funding was available, the campaign coverage would have been more extensive, geographically and impact-wise. Further, we would have been able to conduct an appropriate pre- and post-campaign evaluation of knowledge, attitudes, and practices regarding hygiene, infections, infection control, antibiotic use, antibiotic resistance, and behavior towards these among stakeholders. Public awareness campaigns must be repeated to achieve sustainability in behavioral change. IIMAR wanted to conduct a follow up campaign in 2018 with improvements, but in the absence of supply of funds, a majority of the coordinators expressed an inability to conduct a voluntary campaign once again. It is important to involve stakeholders in animal, agricultural, environmental sectors, etc., in such a campaign, which we could not do. It is not sure, whether this will be easier.

### 4.2. Future Paths

For effective appropriate outcomes from such a campaign, rural areas must be covered, where the inappropriate use of antibiotics is rampant, both by healthcare providers and the population. Antibiotic use is strongly hammered by medical sales representatives causing an increase in its unnecessary use, therefore, these stakeholders must be targeted to reduce inappropriate antibiotic use. Governments have vast infrastructure and resources and also a responsibility for making antibiotic awareness programs successful, therefore government organizations should be instructed to come forward voluntarily to facilitate and support such vital campaigns taken up by non-governmental agencies voluntarily. It needs to be assessed whether bringing forth in campaigns, the harmful side effects of antibiotic misuse, playing on ‘fear for life’ psychological factor among stakeholders, could help in disrupting antibiotics overuse/misuse. Involvement of healthcare provider and patient associations and various types of social and specialist organizations should be explored to increase campaign outreach and effectiveness. Future campaigns also need to integrate and target all types of stakeholders in sectors such as animal, agriculture, environment, manufacturers, pharmaceutical companies, etc., and take up a ‘one health’ approach.

## 5. Conclusions

This appears to be the first antibiotic resistance awareness campaign conducted voluntarily without political and financial support from government that encompasses a vast geographical (3287,590 km^2^) and multi-lingual spread (11 languages) in a single country nationwide, that includes educational information on all aspects of the antibiotic resistance spectrum such as infection control, hand hygiene, appropriate antibiotic use, and antibiotic resistance awareness.

The campaign had a wide product mix and flexible promotion strategy that incorporated multiple pedagogical and participatory physical and electronic techniques for the dissemination of campaign message, which included 33 items (posters, pamphlets, booklets, 11 multilingual audio and 4 bilingual video campaign messages, community radio broadcast, normal radio broadcast, lectures, slogan competition, poster competition, essay competition, awards, lectures, discussion, social media, role play, antibiotic wrappers, Led display, amphitheater display, rallies), with the focus of the campaign components being to adopt the recommended social marketing approach so that the targeted audience owned up to the antibiotic resistance awareness message. In this context, inclusion of community radio stations owned by the community itself, to spread the antibiotic message, needs to be specially mentioned.

As per the recommendations of a covariate analysis that provided theoretical insight into the kind of groups to be targeted for antimicrobial awareness campaigns, we included stewards, stockers, and demanders to achieve antimicrobial stewardship. To achieve this, our campaign covered all sections of society involved in antibiotic prescription, dissemination and use, such as physicians, pharmacists, nurses, hospitals, pathology laboratories, students of medical, pharmacy and nursing colleges, non-medical college students, school students, general public including office goers and small professionals like auto-riksha drivers, spread over cities, towns, district places, and villages.

### Significance and Pointers for Resource Constrained Settings/Low and Middle Income Countries

A successful campaign on antimicrobial/antimicrobial resistance awareness on voluntary basis is feasible and may have an impactful outreach and realistic outcomes as the campaign will be owned by the people doing it, and will not be a part of an imposed/assigned duty. Further, it will be a relatively low cost effort, also easing the burden on the exchequer of resource constrained settings/low and middle income countries.

Lectures appear to be a better way of message spread compared to visuals as lectures are interactive and personal compared to visuals (posters etc.), which are impersonal and their message may not be understood in many places in low income countries where the lay public may not even understand what is an antimicrobial/antibiotic.

A voluntary campaign involving lectures along with discussion will be low cost (US$~1.5). If it is also associated with poster/slogan- display/competition (USD~47), it will help with the understanding and ownership of the campaign message by stakeholders.

Community radio networks are a powerful tool to reach local communities and its involvement will cost the state exchequer very little. Repeated message broadcast on CRS associated with lectures along with discussion and poster/slogan-display/competition during a campaign, appear to help in wide spread assimilation of the message in the public mind.

## Figures and Tables

**Table 1:** Characteristics of the AMRAC-17 campaign in India.

Characteristics	Range	Median
Coordinator’s age (Years)	25–73	40
Number of Co-Coordinators	1–10	2
Number of Volunteers	1–10	3
Expenditure ^1^/Event (INR)(USD) ***	Nil *–50,000 (775)	3000 (47)
Planning and organization ^2^ (days)	1–15	1
Programme (days)/Time (h)	1–7/2–8	4/4
Direct Outreach of the message (Individuals/Event)	50–200,000 **	250
Indirect outreach of the message (Individuals/Event)	10–50,000	500
Positive change in Antibiotic related behavior (Individuals/Event) ^3^	50–4500	100

INR = Indian Rupees, USD = US Dollars. * For a lecture on local conventional radio station (not community radio), the coordinator conveyed expenses to be nil. ** Number indicates estimated listenership for the lecture on radio, *** Exchange rate: 1 USD = 64.5 INR (at the time of campaign). ^1^ The broadcast of the community radio spots in the campaign was gratis. ^2^ AJT and RN spent 3 months and RS, 1 month for planning and coordination, which is not included in calculations. ^3^ Coordinators’ estimate.

**Table 2:** Results of the survey of coordinators, regarding other campaign characteristics.

Characteristic	Yes (%)	No (%)	Maybe (%)
Did you face difficulties in conducting the program?	53	47	0
Will you participate in the campaign again?	84	16	0
Will you be supported again by the same funders?	90	0	10
Did any other agency conduct awareness programs at your place during the AMR week?	5 + 5 *	58	32 ** (Did not know)
Did the campaign create adequate awareness about prudent use of antibiotics?	84	5	11
Did the campaign create adequate awareness about antibiotic resistance?	79	5	16

Note * CME (continuing medical education) in another institute on different dates; ** the answer was ‘did not know’.

## Supplementary Materials

The following are available online at https://www.mdpi.com/1660-4601/16/24/5141/s1; Document S1: Instructions about how to conduct the AMRAC-17 campaign programme; Document S2: Post-campaign questionnaire administered to campaign coordinators to discover campaign characteristics; Figure S1: Examples of Posters used in the campaign and examples from poster making and slogan writing competition; Figure S2: Foldable pamphlets in Hindi, English, Tamil languages used in AMRAC-17 in India; Audio S1: English mini play ‘Take antibiotics as prescribed by a doctor’ broadcast on community radio stations and circulated amongst general public; Video S1: How to perform Alcoholic Hand-rub.
