# Supplementary material for: Characteristics of a Nationwide Voluntary Antibiotic Resistance Awareness Campaign in India; Future Paths and Pointers for Resource Limited Settings/Low and Middle Income Countries
Source: Int J Environ Res Public Health. 2019 Dec 16;16(24):5141. doi: 10.3390/ijerph16245141 (PMC6950494; doi:10.3390/ijerph16245141)
Supplement: Supplementary file 1 [file ijerph-16-05141-s001.zip › Suppl Doc S 1 AMRAC-17 Instructions for Antibiotic Awareness Day procedure.docx]

**Supplementary S1**

**Instructions about how to conduct the AMRAC-17 campaign programme**

**Indian Initiative for Management of Antibiotic Resistance**

**(IIMAR)**

**Antibiotic Resistance Awareness campaign 13-19 November, 2017- AMRAC-17**

**Thank you very much for coming forward to act as coordinator for conducting a programme during the Antibiotic Resistance awareness week - 13-19 November, 2017**

**How the programme can be conducted**

1. Get permission from your University/College/ institute head (Vice Chancellor / Principal/ Director/) to conduct the IIMAR antibiotic resistance awareness program during 13-19 November, 2017 (any one or more days as per your convenience and plan),
2. Form a local committee of your colleagues/respected persons from your institution (even after the program the committee can continue to work for IIMAR). You may invite one or more health related NGO’s in the city to join your campaign
3. The programme can be conducted in several ways/ several modules as per your resources and convenience. We suggest a few here-
4. Arrange lectures, rallies, distribution of brochures/ pamphlets and /or any other thing as per your resources and convenience slogan competition and/ or poster competition, on the topic of  **antibiotic resistance**
5. Content of Lectures could include topics such as knowhow of Antibiotics and Antibiotic resistance, infection control, Hand hygiene, cleanliness, and/or any other relevant topics
6. Invite local microbiologist/pathologist, doctors, pharmacists etc for the programme and ask them to give their experience/views on Antibiotic resistance
7. Please print and distribute the IIMAR antibiotic awareness material (which will be issued by IIMAR) (Distribute as many as you can). If possible release the brochure/pamphlet in front of media persons and get it published in local newspaper**.**
8. If possible, you may **se**lect a public crowded area to distribute the brochures/pamphlets with explanation. You may also distribute brochures in govt./private offices, hospitals / pharmacies /dispensaries )
9. You may use your club’s and chapter’s students related to health or you may use your graduate students to distribute the brochure and explain about antibiotic usage and Antibiotic resistance.
10. Please. print the logo with IIMAR banner and other material supplied by IIMAR
11. If needed please. get prior permission from local police for your activities.
12. Antibiotic resistance awareness is an issue of importance for every individual`s health. If antibiotic resistance spreads, then antibiotics will not be useful in controlling our diseases. So impress this on local people that Antibiotic resistance awareness is very important and generate funds locally for conducting the programme. If IIMAR receives any funds from anywhere, we will try to contribute to your efforts.
