# Supplementary material for: Characteristics of a Nationwide Voluntary Antibiotic Resistance Awareness Campaign in India; Future Paths and Pointers for Resource Limited Settings/Low and Middle Income Countries
Source: Int J Environ Res Public Health. 2019 Dec 16;16(24):5141. doi: 10.3390/ijerph16245141 (PMC6950494; doi:10.3390/ijerph16245141)
Supplement: Supplementary file 1 [file ijerph-16-05141-s001.zip › Suppl Doc S 2 AMRAC-17 Questionnaire for programme coordinators.pdf]

Antimicrobial Resistance Awareness Campaign-AMRAC 13th-19th, Nov 2017 IIMAR`s Nationwide Voluntary Campaign

Questionnaire for program coordinators (IIMAR Nov 2017)

* Required

General

1. Name *

Your answer

Pre-fill responses, then click "Get link"

General

2. Age *

Your answer

3. Qualifications *

Your answer

4. Affiliated institute/ organization *

Your answer

5. Contact details

Your answer

6. Any other information you want to give but not covered earlier
no

Your answer

Funds spent

Pre-fill responses, then click "Get link"

Funds spent for the program? *

Your answer

2. Funded by? *

◯ Coordinator

◯ Institution

◯ Volunteer`s donation

◯ Other:

Others (please mention)

Your answer

2

1. Time spent by coordinator to conduct the program? *

Your answer

Pre-fill responses, then click "Get link"

2. Number of Co -coordinators involved in the program? *

1	2	3	4	5	6	7	8	9	10
○	○	○	○	○	○	○	○	○	○

3. Time spent by Co- coordinators to conduct the program? *

Your answer

4. Number of volunteers involved? *

1	2	3	4	5	6	7	8	9	10
○	○	○	○	○	○	○	○	○	○

5. Time spent by volunteers to conduct the program *

Your answer

Antibiotic Awareness

Pre-fill responses, then click "Get link"

1. In what type of locality did you conduct your programme? *

- ○ City
- ○ District place
- ○ Taluk place
- ○ village

2. Did all your volunteers know that antibiotic resistance is an important health issue? *

- ○ Globally
- ○ Nationally
- ○ Locally
- ○ Other:

3. Did all your co-coordinators know that antibiotic resistance is an important health issue Globally/ Nationally/ Locally? *

- ○ Yes
- ○ No
- ○ Other:

Pre-fill responses, then click "Get link"

3A. If you answered 'no' to the above question, give % not knowing. *

Your answer

4. What were the components of the awareness program and material used for the program? *

- [ ] Oral lecture (self)
- [ ] Oral lecture (invited guest)
- [ ] Distribution of pamphlets (made locally)
- [ ] Distribution of pamphlets (supplied by IIMAR)
- [ ] Display of Posters (made locally)
- [ ] Display of Posters (supplied by IIMAR)
- [ ] Road rally
- [ ] Poster competition
- [ ] Slogan competition
- [ ] Any others

4 A If any other, name the items in short *

Pre-fill responses, then click "Get link"

5. Participants from government - if any, their number? If nil, then say nil *

Your answer

5A. Participants from government- if any, their role?

Your answer

6. Participants from NGO (excluding IIMAR or VIT or DFY)- if any, Name NGO(s) and number of people? if Nil then say nil *

Your answer

6A. Participants from NGO- if any, their role?

Your answer

7. Participants from private organization- if nil say nil, if any give their number? *

Your answer

7A. Participants from private organization- if any, their role?

Pre-fill responses, then click "Get link"

8. To how many people did your message directly reach? Give approximate number in multiple of 50 (like 50, 100 etc) 100 *

Your answer

9. To how many people your message must have Indirectly reached (through the participants talking to others)? Give approximate number in multiples of 50 (like 50, 100 etc). More than 100 *

Your answer

10. Do you think the programme was useful in spreading the awareness about antibiotic use and antibiotic resistance? *

- Yes
- No
- Maybe

Pre-fill responses, then click "Get link"

11. Your estimate how many people may change their behavior regarding antibiotic use due to your programme? *

- ○ Less than 50
- ○ 51-100
- ○ 101-200
- ○ 201-300
- ○ 301-400
- ○ 401-500
- ○ More than 500
- ○ Other:

12. Did you face any difficulties in conducting the program? *

- ○ Yes
- ○ No
- ○ Maybe

If Yes to 12, Describe type of difficulty

Your answer

Pre-fill responses, then click "Get link"

13. How many days did you conduct the programme? *

1	2	3	4	5	6	7	8
○	○	○	○	○	○	○	○

14. Will you conduct such a programme again? *

- ○ Yes
- ○ No
- ○ Maybe

14A If No, give short reason

Your answer

15. Do you think people who supported you in this programme, will support you again? *

- ○ Yes
- ○ No
- ○ Maybe
- ○ If not these, some others may support

Pre-fill responses, then click "Get link"

16. Did any other agency conduct an antibiotic awareness programme in your place? *

○ Yes

○ No

○ Don't know

○ Other:

17 How do you rate your experience of conducting the programme

	1	2	3	4	5
5	○	○	○	○	○

18 Do you feel, the programme created adequate awareness about prudent use of antibiotics

○ Yes

○ No

○ Maybe

Pre-fill responses, then click "Get link"

19 Do you feel, the programme created adequate awareness about antibiotic resistance

- Yes
- No
- Maybe

20 Any suggestions. In rural areas there is very less awareness about MDR bugs. There should be outreach program also in these area, at least to tell people that one should take antibiotics or medicine cautiously.

Your answer

21 I was a participant in the Antibiotic Awareness programme conducted in November 2017 and I give my consent to IIMAR to use the answers given in this form for research or any other purpose connected with the programme.

- Yes
- NO

GET LINK

Never submit passwords through Google Forms.

Pre-fill responses, then click "Get link"

This content is neither created nor endorsed by Google. Report Abuse - Terms of Service

Google Forms

Pre-fill responses, then click "Get link"
