# Supplementary material for: Characteristics of a Nationwide Voluntary Antibiotic Resistance Awareness Campaign in India; Future Paths and Pointers for Resource Limited Settings/Low and Middle Income Countries
Source: Int J Environ Res Public Health. 2019 Dec 16;16(24):5141. doi: 10.3390/ijerph16245141 (PMC6950494; doi:10.3390/ijerph16245141)

Supplementary

Figure S1. Some examples of posters used in the campaign and examples from poster making and slogan writing competition .First row -Hindi language posters regarding antibiotic use. Second row- English and Hindi language posters regarding Hand Hygiene. Third row- One example each from poster making and slogan writing competition. (Posters issued by WHO used in the campaign not shown here)


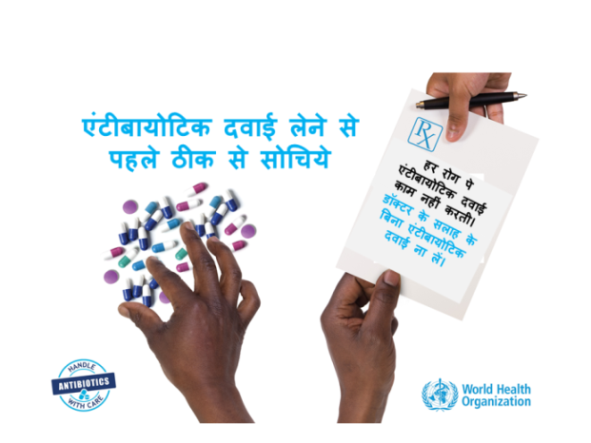

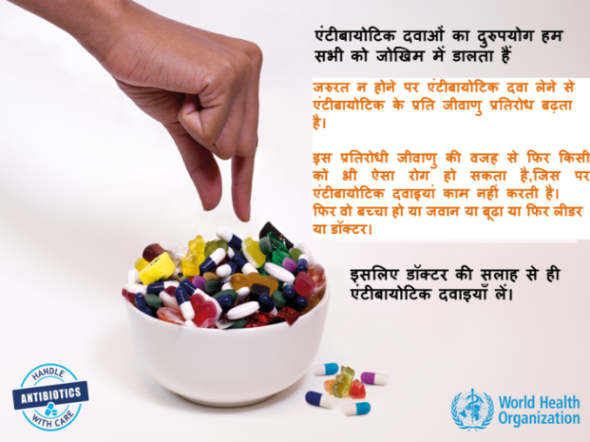

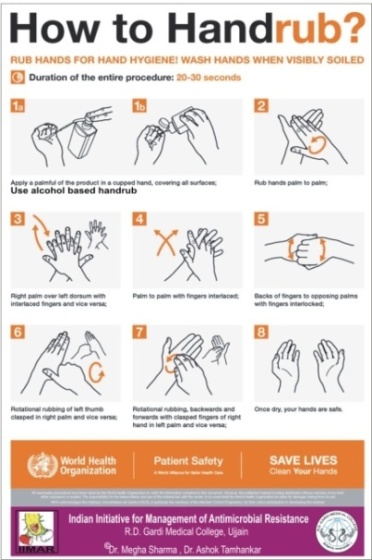

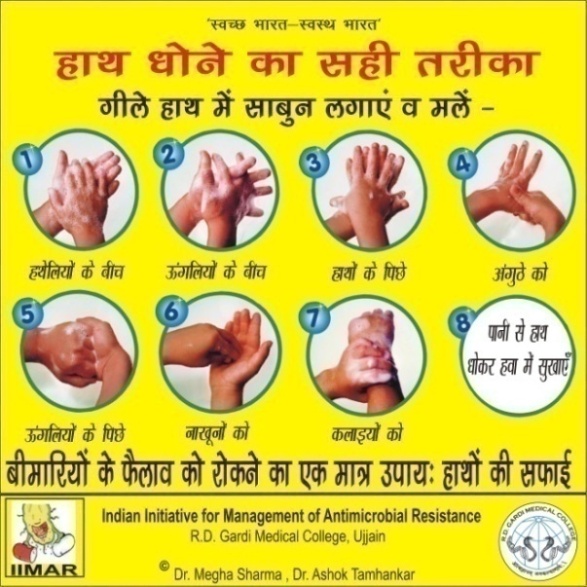

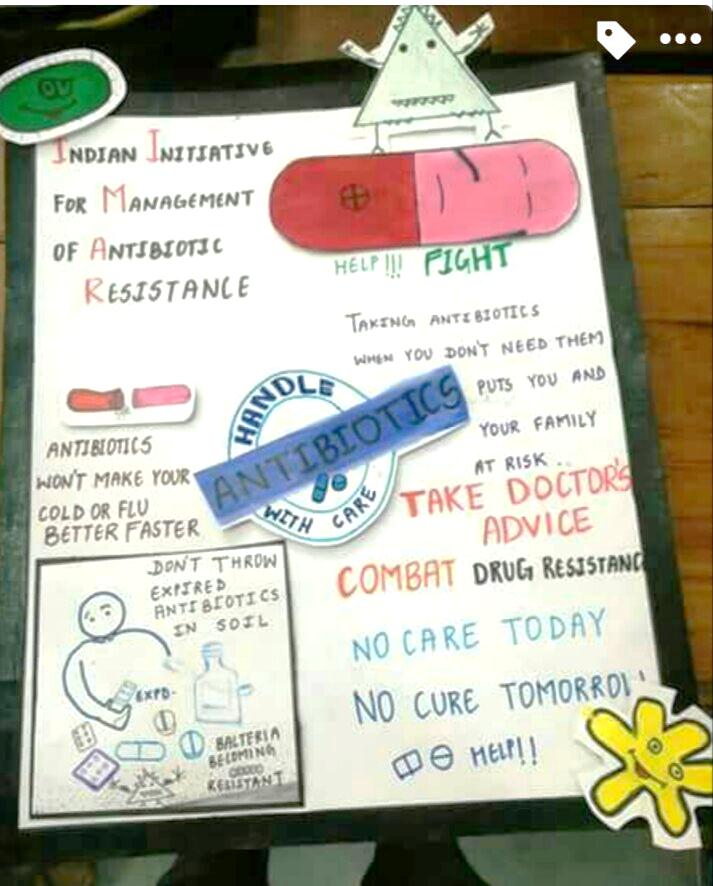

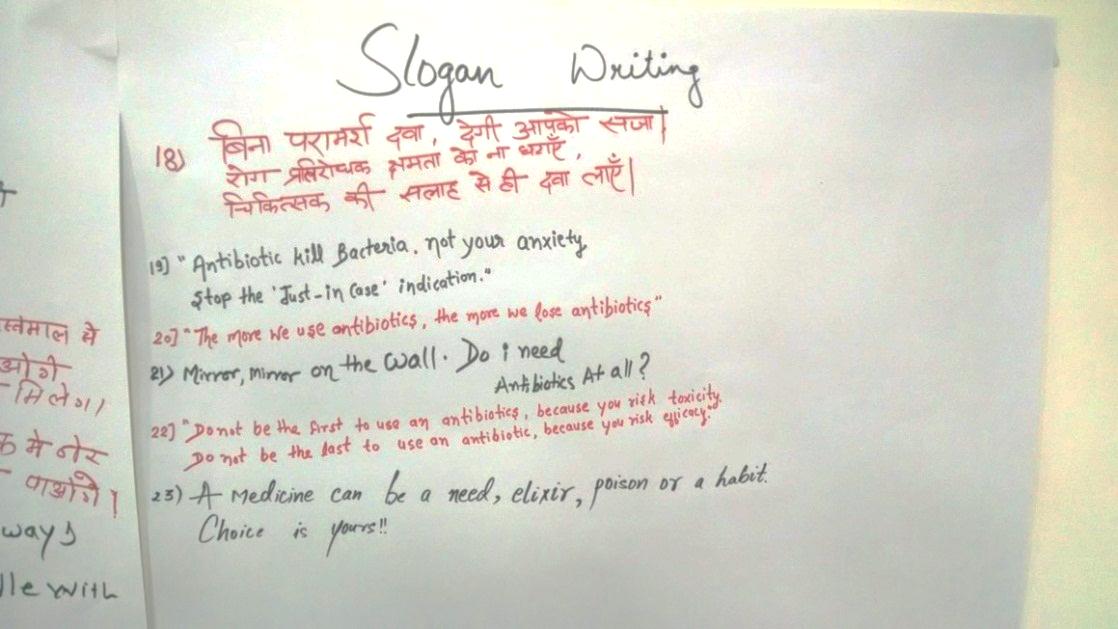

Supplement: Supplementary file 1 [file ijerph-16-05141-s001.zip › Suppl Figure S 1 AMRAC-17 Posters in Campaign.docx]
