# Supplementary material for: Characteristics of a Nationwide Voluntary Antibiotic Resistance Awareness Campaign in India; Future Paths and Pointers for Resource Limited Settings/Low and Middle Income Countries
Source: Int J Environ Res Public Health. 2019 Dec 16;16(24):5141. doi: 10.3390/ijerph16245141 (PMC6950494; doi:10.3390/ijerph16245141)

Supplementary Figure S2. Foldable pamphlets in Hindi, English and Tamil languages used in AMRAC-17 in India. (Front and backside of each) First row - Three fold pamphlet with text in both English and Hindi languages. Second row –Two fold pamphlet in English. Third row –Two fold pamphlet in Tamil language


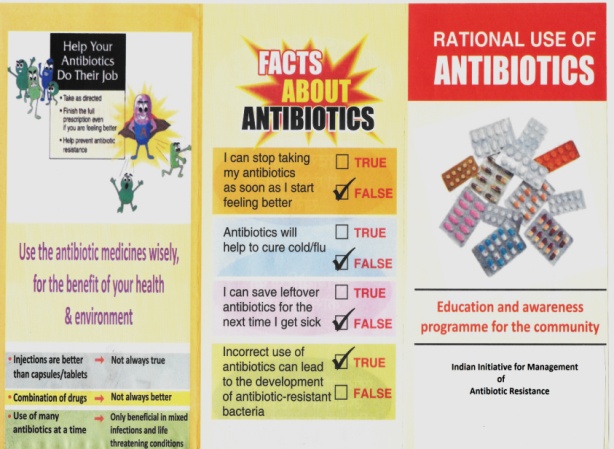

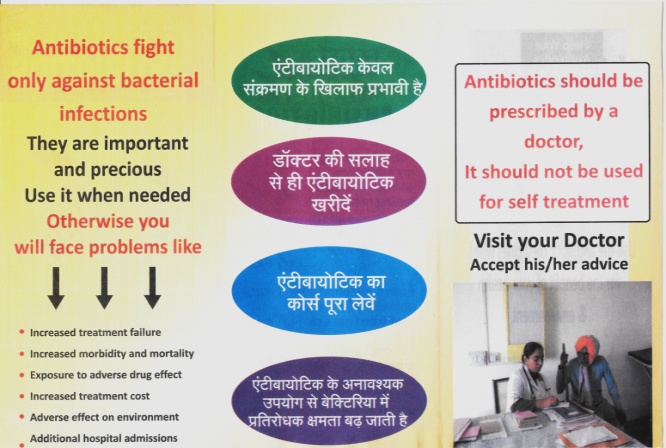

Supplement: Supplementary file 1 [file ijerph-16-05141-s001.zip › Suppl Figure S 2 AMRAC-17 Foldable Pamphlets.docx]
